# Supplementary material for: Translating research into action: Policy recommendations for strengthening antiretroviral therapy adherence in Ghana based on empirical evidence
Source: PLoS One. 2026 May 11;21(5):e0344395. doi: 10.1371/journal.pone.0344395 (PMC13160316; doi:10.1371/journal.pone.0344395)
Supplement: S1 Fig — (DOCX) [file pone.0344395.s001.docx]

**Figure 1: Information Motivation Behaviour Model (Fisher & Fisher, 1992)**





**Figure 2: Health Belief Model (Vitalis, 2017)**
