# Supplementary material for: Translating research into action: Policy recommendations for strengthening antiretroviral therapy adherence in Ghana based on empirical evidence
Source: PLoS One. 2026 May 11;21(5):e0344395. doi: 10.1371/journal.pone.0344395 (PMC13160316; doi:10.1371/journal.pone.0344395)
Supplement: S2 File — (DOCX) [file pone.0344395.s004.docx]

**INTERVIEW GUIDE FOR IN-DEPTH INTERVIEW**

PLHIV/AIDS

1. IDI number:
2. Interviewer’s Name:
3. Note taker’s Name (if available):
4. Location of IDI:
5. Date of IDI (DD/MM/YYYY)
6. Start time:
7. End time:

GENERAL INSTRUCTIONS: Please read the following to the participants. The study team wish to ask you some questions about your experience on adherence to ART. I will ask you questions and my assistant (note-taker) will write down your answers to the questions. We will also audio-record the interview. The interview should take about 20 minutes or little longer to complete. We appreciate your answering these questions as honestly as possible. Please feel free to ask question you don’t understand or feel uncomfortable and we are ever prepare to give you the right response.

Please can we start?

1. **Demographic Information**
2. What is your age in years?
3. What is your sex (M/F)
4. Educational background (please indicate the highest level achieved)
5. What is your religion?
6. What is your marital status?
7. Please are you employed (yes/no)? If yes, what kind of work do you do?
8. **Background**
9. When did you start ART? (Indicate the year and month)
10. How often did you visit the clinic for ART?
11. How did you usually travel to the clinic for treatment? Probe
12. **Experiences of ART adherence**
13. Why did you decide not to adhere to treatment? Can you tell me when that occurred and what informed you to do that.
14. What challenges do you encounter on ART? Probe
15. How did you deal with these challenges? Please give examples.
16. While on treatment, what support systems did you have that helped or not helped you to adhere to ART? Probe
17. Is there anything else you would like to add?

This is the end of the interview. Thank you for your time.
