# Supplementary material for: Translating research into action: Policy recommendations for strengthening antiretroviral therapy adherence in Ghana based on empirical evidence
Source: PLoS One. 2026 May 11;21(5):e0344395. doi: 10.1371/journal.pone.0344395 (PMC13160316; doi:10.1371/journal.pone.0344395)
Supplement: S3 File — (DOCX) [file pone.0344395.s005.docx]

**KEY INFORMANT INTERVIEW GUIDE**

**HEALTH SERVICE PROVIDER**

1. IDI number:
2. Interviewer’s Name:
3. Note taker’s Name (if available):
4. Location of KII:
5. Date of KII (DD/MM/YYYY)
6. Start time:
7. End time:

**GENERAL INSTRUCTIONS**:

Please read the following to the participants. The study team wish to ask you some questions about your experience on adherence to ART. I will ask you questions and my assistant (note-taker) will write down your answers to the questions. We will also audio-record the interview. The interview should take about 20 minutes or little longer to complete. We appreciate your answering these questions as honestly as possible. Please feel free to ask question you don’t understand or feel uncomfortable and we are ever prepared to give you the right response.

Please can we start?

1. **Demographic Information**
2. What is your age in years?
3. What is your sex (M/F)
4. Educational background (please indicate the highest level achieved)
5. What is your religion?
6. What is your marital status?
7. Please are you employed (yes/no)? If yes, what kind of work do you do?
8. Background Information:
9. What type of organization do you work for (if more than one, please describe each one)?
10. How long have you worked for the organization?
11. What role/position do you hold in this organization?
12. Physician or Nurse (c) Pharmacist
13. Counselor (d) Other ……………….
14. **Experiences of ART adherence**
15. Do your patients generally have an understanding of the relationship between HIV and ART?
16. What support systems that you have put in place to encourage clients to adhere to ART?
17. In your opinion, what are the challenges in adhering to ART among HIV/AIDS client.
18. What do you think can be done to improve adherence to ART?
19. Do you have anything else you would like to add?

**This is the end of the interview. Thank you for your time.**
