# Supplementary material for: Translating research into action: Policy recommendations for strengthening antiretroviral therapy adherence in Ghana based on empirical evidence
Source: PLoS One. 2026 May 11;21(5):e0344395. doi: 10.1371/journal.pone.0344395 (PMC13160316; doi:10.1371/journal.pone.0344395)
Supplement: S4 File — (DOCX) [file pone.0344395.s006.docx]

**UNIVERSITY OF CAPE COAST**

**COLLEGE OF EDUCATION STUDIES**

**DEPARTMENT OF HEALTH, PHYSICAL EDUCATION AND RECREATION**

**QUESTIONNAIRE FOR PLHIV/AIDS**

**Dear Respondent**

I am Emmanuel Oduro, a PhD student of the Department mentioned above of the UCC. The objective of the study is to **investigate factors influencing adherence to Anti-Retroviral Therapy among People Living with HIV/AIDS in the Ashanti Region.**

Accepting to take part of this study demands that you complete a prepared question items and semi-structured interview guide that may take about 15 minutes and 25 minutes respectively. You were picked among number of non-adherent to ART to take part of this study and your answers will be analysed as a group. Any participant is taking part of this study on his or her own free will, not coerced and has every right to discontinue without any punishment.

The study has no intention to cause harm to any participant apart from your dedicated time. This is my academic work to ensure a fulfilment of academic requirement. The cost of this study is funded by me and any participant will not be paid for accepting to take part of this study. Your inclusion of this study will help to identify factors influencing adherence to ART among PLHIV/AIDS in the Ashanti Region.

Signature …………………………………… Date ………………………

**SECTION A: Demographic information of participants**

1. Gender: Male [ ] Female [ ]
2. Age in Years: below 15 years [ ] 15-45 [ ] Above 45 [ ]
3. Marital status: single [ ] married [ ] widowed [ ] divorced [ ]
4. Level of education: None [ ] Primary [ ] Secondary [ ] Tertiary [ ]
5. Employment status: employed [ ] unemployed [ ]

**SECTION B: Rate of adherence among the participants**

**Please tick Yes or No in response to the following statements in the table.**

| **Statement** | **Yes** | **No** |
| --- | --- | --- |
| 1. Do you sometimes forget to take your HIV medication? |  |  |
| 1. Do you sometimes find it hard following medical instructions? |  |  |
| 1. Sometimes if you feel sick when you take your HIV medicines do you stop taking it? |  |  |
| 1. When you feel better do you sometimes stop taking your medication? |  |  |
| 1. Do you come on appointment date for drug refill? |  |  |
|  | | |

**SECTION C: Socio-Cultural**

**Instructions: Please tick your response in the table below to indicate the extent to which you strongly agree, agree, disagree or strongly disagree with the following statements.**

| **Statement** | **Strongly Agree** | **Agree** | **Disagree** | **Strongly Disagree** |
| --- | --- | --- | --- | --- |
| 1. HIV can be transmitted from an infected person to another without a curse. |  |  |  |  |
| 1. HIV is a chronic disease. |  |  |  |  |
| 1. Some people get HIV as a punishment for unpleasant behavior. |  |  |  |  |
| 1. Family members and friends can eat together with a person infected with HIV. |  |  |  |  |
| 1. It is important for HIV client disclose his/her status to partner. |  |  |  |  |
| 1. HIV patient needs treatment supporter/monitor. |  |  |  |  |
| 1. HIV patient does not need herbal treatment. |  |  |  |  |
| 1. There is no need to share drugs with an infected partner. |  |  |  |  |

**Please tick Yes or No to show a possible reason why you missed taking your HIV medications (Only for those who missed taking their medicine)**

| **Statement**   1. **I missed taking my HIV medicine because I:** | **Response** | | |
| --- | --- | --- | --- |
|  | **YES** | | **NO** |
| 1. Was away from home |  |  | |
| 1. Wanted to avoid unpleasant feelings that occur when I take the medicine |  |  | |
| 1. Fell asleep at the time I should have taken drug |  |  | |
| 1. Did not feel like taking the HIV pills |  |  | |
| 1. Could not get the medicine from the clinic. |  |  | |
| 1. Used other drugs like herbs |  |  | |
| 1. Was busy with other things |  |  | |
| 1. Don’t have money to travel to clinic |  |  | |
| 1. Don’t have money to eat |  |  | |
| 1. Did not want people to notice me taking drugs |  |  | |
| 1. Forgot |  |  | |
| 1. Drunk alcohol |  |  | |

**SECTION D: Economic factors**

**Please indicate whether you strongly agree, agree, disagree or strongly disagree to the following statements.**

| **Statement** | **Strongly Agree** | **Agree** | **Disagree** | **Strongly Disagree** |
| --- | --- | --- | --- | --- |
| 1. It is difficult for me to get vehicle to the clinic where I receive treatment. |  |  |  |  |
| 1. The distance from where I live to the clinic where I get treatment is long. |  |  |  |  |
| 1. I find it difficult to get food to eat before take medication |  |  |  |  |
| 1. The cost of transportation from where I live to the clinic is high. |  |  |  |  |
| 1. The HIV clinic operating times are not convenient for me. |  |  |  |  |

**SECTION E: Attitude and Behavior of health workers**

**Please indicate whether you strongly agree, agree, disagree or strongly disagree to the following statements.**

| **Statement** | **Strongly Agree** | **Agree** | **Disagree** | **Strongly Disagree** |
| --- | --- | --- | --- | --- |
| 1. I have been educated on the side effects of my HIV drugs and how to manage them. |  |  |  |  |
| 1. Health care providers at the clinic treat me well when I go for treatment. |  |  |  |  |
| 1. Health care providers tell me all I need to know about my HIV disease and treatment. |  |  |  |  |
| 1. I trust the information I received from the provider because my health care providers are competent. |  |  |  |  |
| 1. Health care provider is able to manage all problems I report to the clinic without referring me to another facility. |  |  |  |  |

**SECTION F: Facility support systems**

**Please indicate whether you strongly agree, agree, disagree or strongly disagree to the following statements.**

| **Statement** | **Strongly Agree** | **Agree** | **Disagree** | **Strongly disagree** |
| --- | --- | --- | --- | --- |
| 1. Pharmacy with a trained pharmacist on ARVs is important at the ART clinic. |  |  |  |  |
| 1. ART needs confidential and private location for clinical activities. |  |  |  |  |
| 1. Public health laboratory supports HIV treatment. |  |  |  |  |
| 1. Toilet and sanitary facilities are important at the ART clinic. |  |  |  |  |
| 1. Hospitals’ admission block should not segregated for HIV clients. |  |  |  |  |
