# Supplementary material for: Translating research into action: Policy recommendations for strengthening antiretroviral therapy adherence in Ghana based on empirical evidence
Source: PLoS One. 2026 May 11;21(5):e0344395. doi: 10.1371/journal.pone.0344395 (PMC13160316; doi:10.1371/journal.pone.0344395)
Supplement: S5 File — (DOCX) [file pone.0344395.s007.docx]

**DE-IDENTIFIED QUALITATIVE DATA EXCERPTS AND CODED THEMATIC SUMMARIES**

**Study Title:** Translating Research into Action: Policy Recommendations for Strengthening Antiretroviral Therapy Adherence in Ghana Based on Empirical Evidence

**Data Collection Period:** February 2023 - June 2023
**Location:** Ashanti Region, Ghana
**Participants:** 10 people living with HIV (IDI 001-010) and 5 healthcare providers (KII 001-005)

**THEMATIC ANALYSIS STRUCTURE**

**THEME 1: ECONOMIC BARRIERS TO ADHERENCE**

**Sub-theme 1.1: Transportation Costs and Access Challenges**

**Code:** ECON-TRANSPORT

**Participant Characteristics and Quotes:**

| **Participant ID** | **Demographics** | **Quote** | **Context** |
| --- | --- | --- | --- |
| IDI-001 | Male, 42 years, Primary 4 education, Married, Farmer | "I was experiencing financial difficulties" | Reason for non-adherence |
| IDI-002 | Female, 41 years, JHS education, Married, Employed | "I usually travel with commercial vehicle, but sometimes I walk to the clinic for my drugs" | Transportation method - indicates cost burden |
| IDI-008 | Female, 58 years, Primary 6 education, Divorced, Farmer | "I travelled to take care of my daughter who had delivered a newly born baby. Sometime ago too I was bereaved, I lost my brother so I was not able to come for treatment" | Competing family obligations and transportation barriers |
| KII-002 | Male, 46 years, Masters in Public Health, HIV Coordinator | "I think the clients face socio-economic problems; some clients have lost their job before they were enrolled on ART. They don't have money to pay for transport to access treatment" | Provider perspective on economic barriers |
| KII-005 | Male, 39 years, Degree in Public Health, ART Counselor | "It has been reported that sometimes some clients find it difficult to get money to afford transportation they either walk or fail to come for drugs" | Provider observation on transport cost barriers |

**Coded Summary:**
Transportation costs emerged as a critical economic barrier preventing clinic attendance. Participants described relying on commercial vehicles for clinic visits, with some walking long distances when unable to afford transport. Healthcare providers corroborated that clients frequently missed appointments due to inability to pay for transportation. The barrier was compounded for rural participants and those managing competing family obligations (caregiving, funeral attendance) that required travel funds. Walking to clinics was reported as a coping strategy but created additional time burdens.

**Cross-cutting Factors:**

- Gender: Female participants more frequently mentioned caregiving travel competing with clinic attendance
- Employment status: Unemployed and farming participants most affected
- Rural vs. urban: Rural participants faced longer distances and higher transport costs

**Sub-theme 1.2: Employment Loss and Job Discrimination**

**Code:** ECON-EMPLOY

**Participant Characteristics and Quotes:**

| **Participant ID** | **Demographics** | **Quote** | **Context** |
| --- | --- | --- | --- |
| KII-002 | Male, 46 years, Masters in Public Health, HIV Coordinator | "some clients have lost their job before they were enrolled on ART. They don't have money to pay for transport to access treatment, even some are bread winners who take care of their children can't access ART regularly" | Provider perspective on employment discrimination |
| KII-004 | Female, 30 years, Degree in Psychology, Data Officer | "PLHIV believe that securing a job is their problem because when the employers find out their HIV status they either sack or terminate their appointments" | Provider observation on employment discrimination patterns |
| KII-002 | Male, 46 years, Masters in Public Health, HIV Coordinator | "We tried some time ago to help them, but employers didn't offer them the job due to their HIV status. I suggest the central government should provide them with some jobs to do" | Failed employment linkage attempts |

**Coded Summary:**
HIV-related job loss and employment discrimination created cascading economic barriers. Healthcare providers reported that clients frequently lost employment either before or after ART enrollment due to HIV status disclosure or health deterioration. Attempts by healthcare facilities to link clients with employers failed explicitly due to HIV-related discrimination. Loss of employment not only reduced income for transportation and other expenses but also removed the structure and routine that can support medication adherence. Providers noted that breadwinners experiencing job loss faced particular challenges accessing treatment while managing household responsibilities.

**Cross-cutting Factors:**

- Gender: Both male and female participants affected, but manifestations differed
- Life stage: Breadwinners with dependent children faced compounded challenges
- Disclosure concerns: Fear of employment discrimination prevented status disclosure

**Sub-theme 1.3: Need for Financial and Nutritional Support**

**Code:** ECON-SUPPORT

**Participant Characteristics and Quotes:**

| **Participant ID** | **Demographics** | **Quote** | **Context** |
| --- | --- | --- | --- |
| IDI-001 | Male, 42 years, Primary 4 education, Married, Farmer | "I want financial support to cater for my family and also support my treatment... I need financial support" | Expressed support needs |
| KII-004 | Female, 30 years, Degree in Psychology, Data Officer | "Yes, even a group came to our facility to offer financial support to the PLHIV to start their own jobs and pay back later... Yes our ART unit want financial support and other non-financial assistance such as foodstuffs, clothes, etc" | Microfinance attempts and material needs |
| KII-005 | Male, 39 years, Degree in Public Health, ART Counselor | "Yes I think there should be a system in place to support the clients food though their drugs are being supplied free of charge by Global Fund. I learnt previously they had a system like that to support their treatment" | Past food support programs |

**Coded Summary:**
Participants and providers identified financial and nutritional support as critical adherence enablers. Direct requests for financial assistance emphasized the role of economic stability in supporting treatment engagement. Providers noted that while medications were provided free, lack of food security and basic necessities undermined adherence. Some facilities had experienced microfinance programs, and providers recalled previous food support systems that had been discontinued. Recommendations included restoring nutritional support programs and providing material assistance (food, clothing) alongside medical care.

**Cross-cutting Factors:**

- Economic vulnerability: Farmers and informal sector workers particularly affected
- Family responsibilities: Those supporting dependents emphasized need for family-level support
- Program sustainability: Previous support programs had been discontinued

**THEME 2: HEALTHCARE SYSTEM FACTORS**

**Sub-theme 2.1: Facility Infrastructure and Service Availability**

**Code:** HEALTH-INFRA

**Participant Characteristics and Quotes:**

| **Participant ID** | **Demographics** | **Quote** | **Context** |
| --- | --- | --- | --- |
| IDI-010 | Female, 55 years, MSLC education, Divorced, Palm wine seller | "I want the infrastructure to be expanded such as the lab, pharmacy, counseling room, etc" | Infrastructure improvement needs |
| IDI-008 | Female, 58 years, Primary 6 education, Divorced, Farmer | "I wish the infrastructure here should be improved such as: lab, pharmacy, clinic, etc so that client will spend less time to receive his/her drugs" | Link between infrastructure and waiting times |
| IDI-003 | Male, 44 years, JHS education, Married, Mason | "Yes, I really enjoy the following services: Laboratory services, sanitary system, pharmacy, good counseling, etc" | Positive experience with comprehensive services |
| KII-003 | Female, 48 years, Tertiary education, ART Nurse | "Second challenges are shortages of test kits and pediatric drugs, not frequent but it happens at times" | Supply chain challenges |

**Coded Summary:**
Facility infrastructure limitations directly affected treatment experience and adherence capacity. Participants specifically identified needs for expanded laboratory, pharmacy, and counseling facilities. The link between infrastructure adequacy and waiting times was explicitly recognized - better facilities would enable faster service delivery. Positive experiences were noted when comprehensive services (laboratory, sanitary facilities, pharmacy, counseling) were available in one location. Healthcare providers corroborated infrastructure challenges, noting occasional shortages of test kits and pediatric formulations that complicated care delivery.

**Cross-cutting Factors:**

- Facility location: Government hospitals more commonly cited infrastructure limitations
- Service integration: Participants valued comprehensive services at single location
- Efficiency: Infrastructure adequacy directly affected waiting times

**Sub-theme 2.2: Stigma from Segregated HIV Services**

**Code:** HEALTH-STIGMA

**Participant Characteristics and Quotes:**

| **Participant ID** | **Demographics** | **Quote** | **Context** |
| --- | --- | --- | --- |
| KII-003 | Female, 48 years, Tertiary education, ART Nurse | "the major challenge is the stigmatization, the clients don't feel comfortable visit the ART unit rather they prefer ARVs should be served at their homes without going to the facility" | Preference for home-based services to avoid visible clinic attendance |
| KII-002 | Male, 46 years, Masters in Public Health, HIV Coordinator | "Another challenge is the stigmatization where clients don't want people to notice them as they are coming for their drugs" | Fear of being seen at HIV clinics |
| KII-004 | Female, 30 years, Degree in Psychology, Data Officer | "I think is the stigmatization and discrimination are the main challenges... Stigmatization is a major challenge for PLHIV" | Primacy of stigma as adherence barrier |
| KII-005 | Male, 39 years, Degree in Public Health, ART Counselor | "Stigmatization is the major problem, non-adherent clients think either will meet a relative or friend at the ART unit" | Fear of disclosure through visible service utilization |
| KII-003 | Female, 48 years, Tertiary education, ART Nurse | "I humbly suggest that HIV services should be flexible so that clients can be served with their drugs at home to avoid stigmatization" | Provider recommendation for alternative service delivery |

**Coded Summary:**
Stigmatization related to visible HIV service utilization emerged as a major adherence barrier across participant and provider accounts. Clients expressed discomfort visiting designated ART units due to fear of being identified as living with HIV. The physical separation of HIV services, while intended to provide specialized care, inadvertently created visible markers of HIV status. Participants preferred home-based medication delivery to avoid clinic attendance. Healthcare providers recognized this barrier and recommended flexible service delivery models that would reduce visible identification. Fear of encountering relatives or acquaintances at HIV-specific clinics was explicitly cited as preventing treatment engagement.

**Cross-cutting Factors:**

- Community context: Urban participants expressed greater concerns about being recognized
- Relationship status: Concerns about family and partner disclosure
- Service design: Segregated vs. integrated services created different visibility levels

**Sub-theme 2.3: Healthcare Provider Attitudes and Communication**

**Code:** HEALTH-PROVIDER

**Participant Characteristics and Quotes:**

| **Participant ID** | **Demographics** | **Quote** | **Context** |
| --- | --- | --- | --- |
| KII-001 | Male, 39 years, Second Degree, M&E Officer | "Staff attitude towards clients also play vital role in non-adherence... ability of the health care provider not talk well to the clients, time spent with the clients is a factor, not having enough time to listen to the clients to know their problems. However, always dispensing the ARVs to the clients without listening to them" | Self-awareness of provider communication challenges |
| KII-002 | Male, 46 years, Masters in Public Health, HIV Coordinator | "I think there should be education on stigmatization at the communities and health facilities to accept the HIV clients and support them" | Recognition of facility-level stigma |
| IDI-007 | Female, 44 years, JHS education, Married, Trader | "I wish the ART staff would be in a position to assist the clients in terms of education, counseling, monitoring and drug dispensing all the time" | Desire for consistent provider support |

**Coded Summary:**
Healthcare provider attitudes and communication quality significantly influenced adherence experiences. Providers demonstrated self-awareness about communication challenges, acknowledging that time constraints prevented adequate listening to client concerns. The practice of dispensing medications without substantive engagement was recognized as problematic. Participants valued comprehensive provider support including education, counseling, and monitoring, expressing desire for consistent availability of these services. Providers also identified the need for education within healthcare facilities to reduce provider-level stigma and improve acceptance of people living with HIV.

**Cross-cutting Factors:**

- Resource constraints: Time pressures limited provider-patient interaction quality
- Training needs: Providers identified need for stigma reduction education for healthcare workers
- Service expectations: Participants valued comprehensive counseling and education alongside medication dispensing

**Sub-theme 2.4: Counseling and Support Systems**

**Code:** HEALTH-COUNSEL

**Participant Characteristics and Quotes:**

| **Participant ID** | **Demographics** | **Quote** | **Context** |
| --- | --- | --- | --- |
| IDI-006 | Female, 30 years, No formal education, Cohabiting, Farmer | "I need adherence counseling to be on treatment for a long time without stoppage" | Recognition of counseling importance |
| IDI-009 | Female, 31 years, JHS education, Cohabiting, Trader | "I want treatment monitor, proximity of the pharmacy to enhance effective adherence" | Need for treatment monitoring support |
| KII-001 | Male, 39 years, Second Degree, M&E Officer | "Is to encourage the facilities to engage 'Model of Hopes' and 'Mentor Mothers' currently; the capacity of staffs at the ART clinic to counsel the clients on the ART. Also the follow up calls to reach the clients has been going on" | Peer support and follow-up systems |
| KII-005 | Male, 39 years, Degree in Public Health, ART Counselor | "We have put up a confidential place purposely for counseling" | Counseling infrastructure |

**Coded Summary:**
Counseling and support systems were recognized by both participants and providers as essential adherence enablers. Participants explicitly requested adherence counseling and treatment monitoring to support long-term medication continuation. Peer support mechanisms including "Model of Hopes" and "Mentor Mothers" were being implemented at some facilities. Follow-up phone calls were used to maintain contact with clients. Providers emphasized the importance of confidential counseling spaces and comprehensive support systems (pharmacy proximity, laboratory services, sanitary facilities) as enabling adherence. The quality and consistency of counseling services varied across facilities.

**Cross-cutting Factors:**

- Peer support: Community-based support mechanisms valued
- Service integration: Counseling effectiveness enhanced when integrated with other services
- Confidentiality: Private counseling spaces important for engagement

**THEME 3: SOCIO-CULTURAL FACTORS**

**Sub-theme 3.1: Stigma and Discrimination Experiences**

**Code:** SOCIO-STIGMA

**Participant Characteristics and Quotes:**

| **Participant ID** | **Demographics** | **Quote** | **Context** |
| --- | --- | --- | --- |
| KII-003 | Female, 48 years, Tertiary education, ART Nurse | "the major challenge is the stigmatization, the clients don't feel comfortable visit the ART unit rather they prefer ARVs should be served at their homes without going to the facility" | Avoidance of visible service utilization |
| KII-002 | Male, 46 years, Masters in Public Health, HIV Coordinator | "Another challenge is the stigmatization where clients don't want people to notice them as they are coming for their drugs" | Fear of status disclosure |
| KII-004 | Female, 30 years, Degree in Psychology, Data Officer | "Stigmatization and discrimination are the main challenges... PLHIV believe that securing a job is their problem because when the employers find out their HIV status they either sack or terminate their appointments" | Employment discrimination linked to stigma |
| KII-005 | Male, 39 years, Degree in Public Health, ART Counselor | "Stigmatization is the major problem, non-adherent clients think either will meet a relative or friend at the ART unit" | Anticipated stigma from social networks |

**Coded Summary:**
Stigma and discrimination emerged as pervasive barriers operating at multiple levels. Anticipated stigma (fear of negative reactions) prevented clients from accessing visible HIV services. Enacted stigma manifested through employment discrimination, with employers terminating appointments upon HIV status disclosure. Social stigma concerns centered on fear of encountering relatives or community members at HIV clinics. Internalized stigma contributed to treatment avoidance and reluctance to engage with support systems. Stigma operated at individual, interpersonal, community, and institutional levels, creating compounding barriers to treatment engagement.

**Cross-cutting Factors:**

- Multiple stigma levels: Anticipated, enacted, social, and internalized stigma all present
- Disclosure concerns: Fear of involuntary disclosure through service utilization
- Employment intersection: Stigma directly linked to economic consequences through job discrimination

**Sub-theme 3.2: Cultural Misinformation and Alternative Health Beliefs**

**Code:** SOCIO-MISINFO

**Participant Characteristics and Quotes:**

| **Participant ID** | **Demographics** | **Quote** | **Context** |
| --- | --- | --- | --- |
| IDI-003 | Male, 44 years, JHS education, Married, Mason | "I heard from the radio programme that taking drugs for a long period of time has a negative effect of the body. So I decided to stop taking the drugs for 2 months" | Media misinformation leading to treatment discontinuation |
| KII-001 | Male, 39 years, Second Degree, M&E Officer | "counseling and distortions in messages from health workers, traditional herbalists, pastors, etc on ART" | Multiple sources of conflicting information |

**Coded Summary:**
Cultural information sources significantly influenced treatment decisions, sometimes undermining clinical recommendations. A participant discontinued treatment for two months after hearing radio misinformation about long-term medication effects. Healthcare providers identified multiple sources of conflicting health messages including traditional herbalists and religious leaders alongside inconsistent messages from some healthcare workers. This highlights the influence of culturally circulating health beliefs that may contradict biomedical treatment protocols. The example demonstrates vulnerability to misinformation despite initial treatment engagement and willingness to adhere.

**Cross-cutting Factors:**

- Media influence: Radio programs reached wide audiences with health information
- Alternative medicine: Traditional and spiritual healing systems provided competing frameworks
- Health literacy: Educational level did not fully protect against misinformation influence

**Sub-theme 3.3: Disclosure Challenges and Family Dynamics**

**Code:** SOCIO-DISCLOSE

**Participant Characteristics and Quotes:**

| **Participant ID** | **Demographics** | **Quote** | **Context** |
| --- | --- | --- | --- |
| IDI-008 | Female, 58 years, Primary 6 education, Divorced, Farmer | "I travelled to take care of my daughter who had delivered a newly born baby. Sometime ago too I was bereaved, I lost my brother so I was not able to come for treatment" | Competing family obligations |
| IDI-010 | Female, 55 years, MSLC education, Divorced, Palm wine seller | "I travelled to Accra to offer support to my daughter who had a newly born baby. I was not able to return on time to come for my treatment due to financial difficulties" | Caregiving responsibilities preventing treatment access |
| KII-005 | Male, 39 years, Degree in Public Health, ART Counselor | "non-adherent clients think either will meet a relative or friend at the ART unit" | Fear of family members discovering HIV status |

**Coded Summary:**
Disclosure concerns and family dynamics created complex adherence challenges. Fear of family members discovering HIV status through clinic encounters prevented treatment engagement. Female participants particularly described competing caregiving obligations (supporting daughters after childbirth, funeral attendance for relatives) that prevented clinic attendance. These family responsibilities created both logistical barriers (travel time and costs) and potential disclosure risks. The intersection of caregiving gender roles with HIV status management created particular vulnerabilities for women managing undisclosed status while fulfilling expected family obligations.

**Cross-cutting Factors:**

- Gender roles: Female participants disproportionately described caregiving conflicts
- Life stage: Grandmothers supporting daughters' childbirths particularly affected
- Family structure: Divorced participants managing independent households and extended family obligations

**THEME 4: CLINICAL AND MEDICATION FACTORS**

**Sub-theme 4.1: Adverse Drug Reactions and Side Effects**

**Code:** CLINICAL-ADR

**Participant Characteristics and Quotes:**

| **Participant ID** | **Demographics** | **Quote** | **Context** |
| --- | --- | --- | --- |
| IDI-003 | Male, 44 years, JHS education, Married, Mason | "Yes, I was experiencing adverse drug reaction, but I reported to the prescribers and the drug was substituted" | Successful ADR management through reporting |
| IDI-010 | Female, 55 years, MSLC education, Divorced, Palm wine seller | "I encountered discomfort at the initial stage because I was not following the dosage, instead of 1 daily I was taken 1 morning 1 evening... I came back to the clinic to seek proper education on treatment regimen" | Incorrect dosing due to misunderstanding |

**Coded Summary:**
Adverse drug reactions and medication-related discomfort affected adherence, but responses varied based on healthcare engagement. Successful ADR management occurred when participants reported symptoms and received medication substitution. However, initial treatment discomfort sometimes resulted from dosing misunderstandings rather than pharmacological effects. Participants who sought clarification when experiencing problems generally resolved issues through provider communication. This highlights the importance of clear medication counseling and accessible reporting mechanisms for side effects.

**Cross-cutting Factors:**

- Health literacy: Understanding dosing instructions affected medication experience
- Provider responsiveness: Ability to report and receive medication changes varied
- Treatment education: Quality of initial counseling influenced medication-taking behavior

**Sub-theme 4.2: Misinformation Leading to Treatment Discontinuation**

**Code:** CLINICAL-MISINFO

**Participant Characteristics and Quotes:**

| **Participant ID** | **Demographics** | **Quote** | **Context** |
| --- | --- | --- | --- |
| IDI-006 | Female, 30 years, No formal education, Cohabiting, Farmer | "A midwife told me PMTCT was conducted and my retro status was negative so I decided to stop taking medication. I am not feeling well of late so I decided to come back and continue the treatment" | Healthcare worker miscommunication leading to 2-year treatment discontinuation |
| IDI-009 | Female, 31 years, JHS education, Cohabiting, Trader | "I was misinformed as to when to come for drug refill. The education did not go down well for me. So I was visiting a wrong place for different drugs" | Unclear medication refill instructions |

**Coded Summary:**
Communication failures between healthcare providers and participants led to serious treatment discontinuation. One participant stopped medication for two years after being told her retest was negative during pregnancy, demonstrating how miscommunication can lead to prolonged treatment interruption. Another participant visited incorrect locations for medication refills due to unclear instructions. These examples highlight vulnerabilities created by inadequate or inaccurate information provision from healthcare workers. The consequences included extended periods of viral replication, potential disease progression, and treatment re-initiation challenges.

**Cross-cutting Factors:**

- Provider communication quality: Accuracy and clarity of information varied
- Pregnancy context: PMTCT testing created confusion about treatment continuation
- Health literacy: Participants with lower education particularly vulnerable to miscommunication
- Treatment re-engagement: Recognition of health deterioration prompted return to care

**SYNTHESIS: INTERSECTING BARRIERS**

**Compounding Vulnerabilities**

The qualitative data reveals how economic, healthcare system, and socio-cultural barriers compound to create overwhelming adherence challenges:

1. **Economic-Stigma Intersection:** Fear of HIV status disclosure prevents treatment engagement, while HIV-related job discrimination creates economic hardship that further limits ability to access treatment. Loss of employment reduces funds for transportation while simultaneously increasing time available (but not financial means) for clinic attendance.
2. **Healthcare System-Stigma Intersection:** Segregated HIV services intended to provide specialized care inadvertently create visible markers of HIV status, reinforcing stigma and preventing service utilization. This creates a paradox where privacy-enhancing segregation actually undermines treatment engagement through increased visibility.
3. **Gender-Economic-Caregiving Intersection:** Female participants experienced compounded challenges where caregiving responsibilities (culturally expected gender roles) competed with treatment access, requiring both travel funds and time away from income-generating activities. Divorced or separated women managing independent households faced particular challenges balancing economic survival, family obligations, and treatment adherence.
4. **Age-Employment-Disclosure Intersection:** Younger participants expressed greater concerns about employment discrimination and identity management, while older participants more commonly described integration of treatment into established life routines but faced challenges with family caregiving obligations.

**Protective Factors**

Several protective factors emerged that facilitated adherence despite barriers:

1. **Comprehensive Facility Services:** Participants valued facilities offering integrated services (laboratory, pharmacy, counseling, sanitary facilities) at single locations, reducing required visits and time investment.
2. **Responsive Provider Communication:** When participants reported problems (adverse drug reactions, dosing confusion) and received appropriate responses (medication changes, clarified instructions), adherence improved.
3. **Peer Support Mechanisms:** "Model of Hopes" and "Mentor Mothers" programs provided community-based support that reduced isolation and offered practical adherence assistance.
4. **Flexible Service Delivery:** Multi-month dispensing reduced required clinic visits, particularly benefiting employed participants and those in rural areas.

**Policy Implications**

The qualitative findings strongly support the need for:

1. **Integrated Service Delivery:** Reducing visible identification through non-segregated HIV services while maintaining privacy through private consultation spaces within general healthcare settings.
2. **Economic Support Packages:** Addressing transportation costs, employment discrimination, and nutritional needs simultaneously rather than treating these as separate concerns.
3. **Provider Training:** Improving communication quality, reducing provider-level stigma, and ensuring accurate information provision to prevent miscommunication-driven treatment discontinuation.
4. **Community Engagement:** Proactively addressing health misinformation in community media and cultural contexts rather than relying solely on individual counseling after adherence problems emerge.

**METHODOLOGICAL NOTES**

**Sample Characteristics:**

- **People living with HIV (n=10):** Ages 30-58 years; 6 female, 4 male; education ranged from no formal education to JHS; employment included farming, trading, mason work; marital status varied (married, divorced, cohabiting, single)
- **Healthcare providers (n=5):** Ages 30-48 years; 2 female, 3 male; education ranged from first degree to Masters; roles included HIV coordinators, ART nurses, counselors, data officers, M&E officers

**Interview Duration:** 10-30 minutes per interview

**Data Collection:** Semi-structured interviews conducted in private rooms within healthcare facilities; interviews audio-recorded and transcribed

**Thematic Analysis Approach:** Inductive coding with iterative refinement through research team discussion; themes organized to align with theoretical frameworks (Information-Motivation-Behavioral Skills Model and Social Ecological Model)

**Ethical Considerations:** All identifying information removed; participant IDs assigned for tracking while preserving anonymity; quotes selected to represent themes without compromising confidentiality
