# Supplementary material for: Translating research into action: Policy recommendations for strengthening antiretroviral therapy adherence in Ghana based on empirical evidence
Source: PLoS One. 2026 May 11;21(5):e0344395. doi: 10.1371/journal.pone.0344395 (PMC13160316; doi:10.1371/journal.pone.0344395)
Supplement: Attachment — Submitted filename: Reviever comments.doc [file pone.0344395.s008.doc]

Manuscript titled - Translating Research into Action: Policy Recommendations for Strengthening Antiretroviral Therapy Adherence in Ghana Based on Empirical Evidence, is a very well written article resulting from detailed scientific and methodical research work.

Recommendation- Can be accepted for publication.
