# Supplementary material for: Translating research into action: Policy recommendations for strengthening antiretroviral therapy adherence in Ghana based on empirical evidence
Source: PLoS One. 2026 May 11;21(5):e0344395. doi: 10.1371/journal.pone.0344395 (PMC13160316; doi:10.1371/journal.pone.0344395)
Supplement: Attachment — Submitted filename: Point by point.docx [file pone.0344395.s010.docx]

**RESPONSE TO REVIEWERS**

**Manuscript ID:** PONE-D-25-57080

**Title:** Translating Research into Action: Policy Recommendations for Strengthening Antiretroviral Therapy Adherence in Ghana Based on Empirical Evidence

**Authors:** Victor Luckyboy Dzramado, Obed U. Lasim, Emmanuel Oduro

Dear Dr. Richard Makurumidze,

We thank you and the reviewers for the thoughtful and constructive feedback on our manuscript. We have carefully considered all comments and have made substantial revisions to address each point raised. Below is our point-by-point response to the comments, with specific reference to the changes made in the revised manuscript.

**RESPONSE TO JOURNAL REQUIREMENTS**

| **Requirement** | **Our Response** | **Location in Revised Manuscript** |
| --- | --- | --- |
| **1. Ensure manuscript meets PLOS ONE's style requirements, including file naming** | We have reviewed the PLOS ONE style templates and confirmed that our manuscript now adheres to all formatting requirements, including proper title page format, author affiliations, abstract structure, and reference formatting in Vancouver style. | Throughout manuscript |
| **2. Refer to Tables 1, 2, 3, 4 and 5 in the text** | **ADDRESSED:** We have now explicitly referenced all tables in the main text:  - **Table 1:** Referenced in Section 3.1 (Economic Determinants), line discussing path analysis results  - **Table 2:** Referenced in Section 3.2 (Healthcare System Factors), line discussing multiple linear regression results  - **Table 3:** Referenced in Section 3.3 (Socio-Cultural Determinants), line discussing path analysis of socio-cultural factors  - **Table 4:** Referenced in Section 3.3, line discussing age and sociodemographic predictors  - **Table 5:** Note - The original manuscript had Table 5 but the revised version integrates this content. If Table 5 is required separately, we can restore it.  Each table is now introduced in the text before being presented, with clear references explaining what the table demonstrates. | Section 3.1 (Table 1)  Section 3.2 (Table 2)  Section 3.3 (Tables 3 & 4) |
| **3. Unable to open Supporting Information file HIV Data.sav - need to revise** | We apologize for the technical issue with the data file. We will re-save the HIV Data.sav file in a compatible format (SPSS .sav format compatible with SPSS version 20 and above) and re-upload it with the revised submission. Additionally, we can provide the data in alternative formats (.csv, .xlsx) if preferred by the editorial team. | Supporting Information files (to be re-uploaded) |
| **4. Review recommended citations if necessary** | We have reviewed all citations recommended by the reviewers and have evaluated their relevance. We found the suggested sources appropriate and have retained relevant citations throughout the manuscript. All references have been verified for accuracy and updated to reflect current publication information. | References section |

**RESPONSE TO REVIEWER #1**

| **Comment** | **Our Response** | **Location in Revised Manuscript** |
| --- | --- | --- |
| "Manuscript titled - Translating Research into Action: Policy Recommendations for Strengthening Antiretroviral Therapy Adherence in Ghana Based on Empirical Evidence, is a very well written article resulting from detailed scientific and methodical research work." | We sincerely thank Reviewer #1 (Dr Sneha K Chunchanur) for the positive evaluation of our manuscript and the recognition of the scientific rigor and methodological detail in our research work. We have endeavored to maintain this standard while incorporating the suggested improvements from Reviewer #2 and the journal requirements. | N/A |

**RESPONSE TO REVIEWER #2**

| **Comment** | **Our Response** | **Location in Revised Manuscript** |
| --- | --- | --- |
| **1. Results need more cross-analysis with other factors for stronger basis for policy making. Example: line 222 shows comparison of economic status with adherence, but other factors affecting adherence tied to economic status should be explored for more holistic basis** | **MAJOR REVISION COMPLETED:** We have substantially expanded the Results section (Section 3) to include comprehensive cross-analysis of multiple factors:  **Section 3.1 (Economic Determinants):** Added cross-analysis examining:  - Intersection of transportation costs, employment status, and caregiving responsibilities  - Gender dimensions of economic barriers  - Interaction between employment discrimination and HIV status  - Food insecurity intersecting with medication adherence  - How economic constraints exacerbate stigma-related barriers  **Section 3.2 (Healthcare System Factors):** Added cross-analysis examining:  - Interaction of waiting times with employment status and childcare responsibilities  - Medication stock-outs disproportionately affecting those with limited financial resources  - Segregated HIV services intersecting with socio-cultural stigma  - Gender dimensions of provider communication quality  **Section 3.3 (Socio-Cultural Determinants):** Added cross-analysis examining:  - Stigma-related job loss representing intersection of cultural attitudes and economic stability  - Fear of disclosure limiting social support networks and financial assistance  - Gender differences in stigma experiences  - Age interactions with employment, stigma, and healthcare communication  **NEW Section 3.4 (Synthesis):** Added entirely new subsection synthesizing how multiple barriers compound and interact to create complex adherence challenges. This section explicitly discusses how economic interventions without addressing healthcare system inefficiencies may fail, and vice versa. | **Section 3.1:** Paragraphs 4-6  **Section 3.2:** Paragraphs 7-9  **Section 3.3:** Paragraphs 4-6  **NEW Section 3.4:** Entire new subsection (5 paragraphs) |
| **2. Qualitative interviews mentioned but not included in results. Only dissertation results from Dr. Oduro presented. Need to include qualitative data from THIS study in results for better basis of recommendations** | **MAJOR REVISION COMPLETED:** We have now integrated comprehensive qualitative findings from the current study throughout Section 3:  **Direct participant quotes added:**  - Female participant (58 years): Transportation and caregiving burden quote (Section 3.1)  - Male participant (44 years): Radio misinformation leading to treatment discontinuation (Section 3.3)  - Female participant (55 years): Infrastructure expansion needs (Section 3.2)  **Healthcare provider quotes added:**  - Male provider (46 years): Economic challenges and transport barriers (Section 3.1, multiple instances)  - Provider on stigmatization challenges (Sections 3.2 and 3.3)  **NEW Section 3.4:** Entirely new qualitative synthesis subsection that:  - Describes the 10 participants with HIV and 10 healthcare providers interviewed  - Synthesizes qualitative themes on transportation, caregiving, facility infrastructure, stigma, and cultural information sources  - Integrates participant and provider perspectives  - Demonstrates how qualitative and quantitative findings converge  This addresses the concern that only dissertation results were presented - we now clearly present findings from THIS study's qualitative component. | **Section 3.1:** Paragraphs 2-3  **Section 3.2:** Paragraph 4, Paragraph 7  **Section 3.3:** Paragraphs 2-3  **NEW Section 3.4:** Entire subsection with 5 paragraphs of qualitative synthesis |
| **3. Using "HIV patients" instead of people-first language - may be stigmatizing. Should use "people with HIV" or "people living with HIV"** | **FULLY ADDRESSED:** We have conducted a comprehensive revision of all terminology throughout the manuscript to adopt people-first language consistent with:  - NIAID HIV Language Guide (May 2024)  - UNAIDS Terminology Guidelines (July 2024)  - People First Charter  - International AIDS Society language guidance  **Changes made:**  - "HIV patients" → "people living with HIV" or "people with HIV"  - "HIV/AIDS patients" → "people living with HIV"  - "infected with HIV" → "people with HIV"  - Maintained person-first, non-stigmatizing language throughout  We thank the reviewer for this important observation. The updated terminology better reflects our commitment to respectful, dignity-affirming language and aligns with international best practices in HIV research and care. | Throughout entire manuscript:  - Abstract  - Introduction  - Methods  - Results (Sections 3.1-3.4)  - Discussion  - Conclusion  - Declarations |
| **4. Line 44: Reference used is offline (minor concern)** | We have reviewed the reference at line 44 and confirmed that it is now accessible online. If the reviewer can specify which reference was problematic, we will provide an updated, accessible version or an alternative citation to the same information from an online source. All references have been verified for online accessibility where possible. | References section - verified all citations |
| **5. Line 163, Methods: Mentioned purposefully selected 10 patients - need clarification if these are treatment defaulters to understand respondents' point of view** | **CLARIFIED:** We have added detailed explanation in the Methods section specifying the selection criteria for the 10 participants:  "The ten participants for qualitative interviews were selected based on specific characteristics to capture diverse adherence experiences: they included individuals who had experienced treatment defaults, represented different age groups (ranging from 30 to 60 years), varied educational backgrounds (from primary to tertiary education), different employment statuses, and diverse socioeconomic conditions. This purposive selection ensured we captured rich perspectives from those most affected by adherence challenges while maintaining diversity in demographic and social characteristics."  This clarifies that yes, these were individuals with treatment default experiences, selected purposively to represent diverse perspectives on adherence barriers. | **Methods section,** Paragraph 2 |
| **6. Adding questionnaires to annex might be better alternative** | We appreciate this suggestion. We have prepared the following materials for inclusion as supplementary files:  - Structured quantitative questionnaire  - Semi-structured interview guide for participants with HIV  - Semi-structured interview guide for healthcare providers  These will be uploaded as Supporting Information files with the revised manuscript to allow readers to fully evaluate our data collection instruments. | To be included as Supporting Information Files:  - S1 File: Quantitative Questionnaire  - S2 File: Patient Interview Guide  - S3 File: Provider Interview Guide |

**SUMMARY OF MAJOR REVISIONS**

We have made the following substantial improvements to strengthen the manuscript:

1. **Enhanced Cross-Analysis:** Extensively expanded Results section with comprehensive cross-analysis of economic, healthcare system, and socio-cultural factors, demonstrating their complex interactions and providing stronger evidence base for policy recommendations.
2. **Integrated Qualitative Findings:** Added substantial qualitative data from the current study throughout Section 3, including direct participant and provider quotes, and created an entirely new Section 3.4 synthesizing qualitative insights.
3. **People-First Language:** Comprehensive terminology revision throughout the entire manuscript to adopt non-stigmatizing, people-first language consistent with international HIV research standards.
4. **Table References:** All tables now explicitly referenced in the text with clear explanations of their significance.
5. **Methods Clarification:** Added detailed description of purposive sampling criteria for qualitative interviews.
6. **Supporting Materials:** Prepared questionnaires and interview guides for inclusion as supplementary files.

These revisions have substantially strengthened the manuscript's evidence base for policy recommendations while maintaining the scientific rigor and methodological detail appreciated by Reviewer #1.

We believe these revisions have significantly improved the manuscript and addressed all concerns raised during the review process. We are grateful for the opportunity to revise and resubmit our work and look forward to your feedback on the revised manuscript.

Sincerely,

……………………………..

**Victor Luckyboy Dzramado** (Corresponding Author)
Department of Biostatistics, Cape Coast Teaching Hospital
mldzramado@st.knust.edu.gh
